# Cyclic GMP-AMP Triggers Asthma in an IL-33-Dependent Manner That Is Blocked by Amlexanox, a TBK1 Inhibitor

**DOI:** 10.3389/fimmu.2019.02212

**Published:** 2019-09-26

**Authors:** Koji Ozasa, Burcu Temizoz, Takato Kusakabe, Shingo Kobari, Masatoshi Momota, Cevayir Coban, Shuichi Ito, Kouji Kobiyama, Etsushi Kuroda, Ken J. Ishii

**Affiliations:** ^1^Laboratory of Adjuvant Innovation, Center for Vaccine and Adjuvant Research, National Institutes of Biomedical Innovation, Health and Nutrition, Osaka, Japan; ^2^Department of Pediatrics, Yokohama City University Graduate School of Medicine, Yokohama, Japan; ^3^Laboratory of Vaccine Science, WPI Immunology Frontier Research Center, Osaka University, Osaka, Japan; ^4^Division of Vaccine Science, Department of Microbiology and Immunology, The Institute of Medical Science, The University of Tokyo, Tokyo, Japan; ^5^International Research and Development Center for Mucosal Vaccines, The Institute of Medical Science, The University of Tokyo, Tokyo, Japan; ^6^Laboratory of Mock-Up Vaccine Project, Center for Vaccine and Adjuvant Research, National Institutes of Biomedical Innovation, Health and Nutrition, Osaka, Japan; ^7^Laboratory of Malaria Immunology, WPI Immunology Frontier Research Center, Osaka University, Osaka, Japan; ^8^Division of Malaria Immunology, Department of Microbiology and Immunology, The Institute of Medical Science, The University of Tokyo, Tokyo, Japan; ^9^Department of Immunology, Hyogo College of Medicine, Nishinomiya, Japan

**Keywords:** asthma, cGAMP, house dust mite, IL-33, TBK-1 inhibitor

## Abstract

Extracellular host-derived DNA, as one of damage associated molecular patterns (DAMPs), is associated with allergic type 2 immune responses. Immune recognition of such DNA generates the second messenger cyclic GMP-AMP (cGAMP) and induces type-2 immune responses; however, its role in allergic diseases, such as asthma, has not been fully elucidated. This study aimed to determine whether cGAMP could induce asthma when used as an adjuvant. We intranasally sensitized mice with cGAMP together with house dust mite antigen (HDM), followed by airway challenge with HDM. We then assessed the levels of eosinophils in the broncho-alveolar lavage fluid (BALF) and serum HDM-specific antibodies. cGAMP promoted HDM specific allergic asthma, characterized by significantly increased HDM specific IgG1 and total IgE in the serum and infiltration of eosinophils in the BALF. cGAMP stimulated lung fibroblast cells to produce IL-33 *in vitro*, and mice deficient for IL-33 or IL-33 receptor (ST2) failed to develop asthma enhancement by cGAMP. Not only *Il-33*^−/−^ mice, but also *Sting*^−/−^, *Tbk1*^−/−^, and *Irf3*^−/−^*Irf7*^−/−^ mice which lack the cGAMP-mediated innate immune activation failed to increase eosinophils in the BALF than that from wild type mice. Consistently, intranasal and oral administration of amlexanox, a TBK1 inhibitor, decreased cGAMP-induced lung allergic inflammation. Thus, cGAMP functions as a type 2 adjuvant in the lung and can promote allergic asthma in manners that dependent on the intracellular STING/TBK1/IRF3/7 signaling pathway and the resultant intercellular signaling pathway via IL-33 and ST2 might be a novel therapeutic target for allergic asthma.

## Introduction

Asthma is a common chronic respiratory illness with an increasing prevalence especially in developed countries (1, 2). Asthma may consist of many phenotypes based on the onset of disease (3, 4). Of these phenotypes, early-onset asthma during childhood is mainly associated with type 2 immune responses. Although many factors, such as infection, environmental factors, and genetic factors, contribute to the onset of asthma (5), the mechanism by which type 2 immune responses are activated remains elusive.

We previously reported that alum induced cell death and that the host DNA released from dying cells has an important role in the production of IgE (6). Additionally, other work demonstrated that host-derived DNA is recognized as a damage-associated molecular pattern (DAMP) and induces type 2 immune responses and allergic inflammation (7). Moreover, it was reported that host DNA released by NETosis promote type-2 allergic asthma exacerbation (8). However, the mechanisms responsible for the recognition of this DNA and the subsequent induction of immune responses are not fully understood.

Cyclic GMP-AMP (cGAMP) has received much attention as a second messenger following recognition of intracellular DNA and virus infection (9). DNA from pathogens is recognized by cyclic GMP-AMP synthase (cGAS), and the resulting enzymatic activity of cGAS generates cGAMP using ATP and GTP as substrates. Several studies have shown that host DNA release is observed at the site of inflammation (10). Interestingly, the released DNA seems to be derived not only from dying or stressed cells but also from neutrophils and eosinophils as a result of their activation. Some studies have suggested that this DNA can be recognized by cytosolic cGAS and recruit stimulator of interferon (IFN) genes (STING) at plasma membrane, which in turn activate, and is phosphorylated by, Tank binding kinase 1 (TBK1), a non-canonical IKK, followed by the transcription factor of IRF3 and IRF7, turning on many IFN-inducible genes (11). Furthermore, we previously found that cGAMP and other STING ligands are strong inducers of antigen-specific type 2 immune response via a type-I IFN-dependent pathway (12, 13). These findings suggest that DNA release at the site of inflammation, such as during infection, is associated with the activation of type 2 immune responses and, additionally, that cGAMP is associated with this activation as a mediator.

Here, we assessed the possibility that cGAMP can act as an allergic adjuvant by coadministration of intra-nasal cGAMP with house dust mite antigen (HDM). We used cGAMP as an alternative stimulus for DNA, because cGAS binds dsDNA and generates the second messenger cGAMP.

## Materials and Methods

### Reagents

The 2′-3′ and 3′-3′ cGAMP were purchased from InvivoGen (San Diego, CA, USA). HDM extracts (Mite Extrac-Df; LG5339, extract from house dust mites, *Dermatophagoides farinae*, 1.7 μg endotoxin/mg) were purchased from LSL (Osaka, JAPAN). Amlexanox was purchased from the Tokyo Chemical Industry and Takeda Pharmaceutical Company (Osaka, Japan). Dexamethasone was purchased from Sigma Aldrich (St. Louis, MO, USA).

### Mice

Six-week-old female C57BL/6J mice were purchased from CLEA Japan Inc. (Osaka, Japan). *Sting*^−/−^ mice were purchased from Phoenix Bio (Hiroshima, Japan). *Il-33*^−/−^, *Rag2*^−/−^, and *St-2*^−/−^ mice were obtained as previously described (14–16). *Tslp*^−/−^ mice were kindly provided by Dr. Ziegler (Immunology Program, Benaroya Research Institute, Seattle, WA, USA). *Irf7*^−/−^ mice were provided by the RIKEN BioResource Center through the National Bio-Resource Project of the Ministry of Education, Culture, Sports, Science and Technology (Ibaraki, Japan) (17). *Irf3*^−/−^*Irf7*^−/−^ mice were generated by cross-breeding *Irf3*^−/−^ mice with *Irf7*^−/−^ mice, and *Tnf* ^−/−^*Tbk1*^−/−^ mice were obtained as previously described (18). All animal experiments were conducted in accordance with the institutional guidelines and with the approval of the ethics committee for the National Institute of Biomedical Innovation, Health and Nutrition (NIBIOHN, Approval ID: DNA301).

### Mouse Model of cGAMP-Adjuvanted, HDM-Induced Airway Inflammation

Mice were anesthetized by ketamine and xylazine followed by the intranasal administration of 1 μg HDM, 1 μg cGAMP, or HDM + cGAMP in 30 μl of PBS at day 0, and then challenged with 1 μg HDM four times (at days 7, 9, 11, and 13). Samples [serum, broncho-alveolar lavage fluid (BALF), lymph nodes, and lungs] were collected 24 h after the last HDM challenge.

### Viruses and Infection

Stocks of influenza virus (A/PR8/34) were grown in Madin-Darby canine kidney cells. Mice were infected intranasally with 140 plaque-forming units of influenza virus. Stocks of purified RSV (strain A2, ATCC) were grown in HEp-2 cells. Inactivation was performed by heating the virus to 56°C for 30 min. For infection, mice were anesthetized, and an intratracheal instillation with 2.5 × 10^5^ plaque-forming units of RSV was performed.

### *In vitro* Stimulation of Lung Fibroblast Cells

To obtain suspensions of single lung cells, mouse lungs were removed, minced, and digested in RPMI 1640 with 200 μg/ml DNase (Roche, Penzberg, Germany) and 200 units/ml collagenase (Wako, Osaka, Japan) and then incubated at 37°C for 1 h. The lungs were homogenized with the gentleMACS™ Dissociator (Miltenyi Biotech, Gladbach, Germany), and debris was removed by using a 70-μm separation filter. The cells were washed twice with phosphate-buffered saline (PBS) and then resuspended in Dulbecco's modified Eagle's medium with 10% fetal calf serum and 5 μg/ml of insulin and seeded into 75 cm^2^ culture flasks (Corning, Corning, NY, USA). Medium was replaced after 24 h, and then every 2 days. Adherent cells were used as lung fibroblast cells. These cells were incubated with cGAMP (1 μg/ml or 10 μg/ml) for 6, 24, 48 h, after the cytokine content of the resulting cell lysates were measured. Cell lysates were prepared with three freeze-thaw cycles.

### BALF Collection and Cell Population Assessment

Broncho-alveolar lavage fluid was collected by two lung lavages with 0.7 ml of PBS each via a tracheal cannula. The collected BALF was centrifuged (2,000 × *g*, 5 min, 4°C) to separate the cells and fluids, and then the cells were counted in a hemocytometer. Next, the cells were incubated with anti-mouse CD16/32 antibody, and then stained with anti-CD11c, anti-CD11b, anti-Siglec F, anti-F4/80, anti-Ly6G, anti-CD3, and anti-B220 antibodies. All labeled antibodies were purchased from BD Biosciences or BioLegend. The samples were analyzed by flow cytometry (LSRII, BD Biosciences, Franklin Lake, NJ, USA). The resulting data were analyzed using FlowJo software.

### Lymph Node Assays

Single-cell suspensions of bronchial lymph nodes (2 × 10^5^ cells in a 96-well plate) were cultured in RPMI (containing 10% fetal calf serum and 1% penicillin/streptomycin) with or without HDM (10 μg/ml) for 5 days. Enzyme-linked immunosorbent assays (ELISAs) (described below) were used to assess the IL-5 and IL-13 levels in the supernatants.

### Measurement of Antibodies and Cytokines

To measure the HDM-specific IgG1 levels, serial dilutions of sera were prepared in 96-well plates coated with 10 μg/ml of HDM. Horseradish peroxidase-conjugated anti-mouse IgG1 (Southern Biotech, Birmingham, AL, USA) was used as the secondary antibody. The total IgE was measured by a mouse IgE ELISA Quantitation kit (Bethyl Laboratories Inc., Montgomery, TX, USA) according to the manufacturer's protocol. Cytokines (IL-5 and IL-13) were quantified by ELISA kits (R&D Systems, Minneapolis, MN, USA) according to the manufacturer's protocols.

### Measurement of Double-Stranded (ds)DNA

The double-stranded (ds)DNA concentrations were measured by using Qbit dsDNA HS Assay kits (InvitroGen, Carlsbad, CA) according to the manufacturer's protocol.

### Histological Analyses

Lung tissues were dissected and fixed in 4% paraformaldehyde overnight. To prepare the paraffin sections, tissues were gradually dehydrated and embedded in paraffin. The sections (5 μm thick) were prepared and stained with hematoxylin and eosin (H&E) or periodic acid-Schiff (PAS) using a Periodic Acid-Schiff kit (Sigma Aldrich) according to the manufacturer's protocol. Representative images were acquired with a ScanScope® AT slide scanner (Leica, Wetzlar, Germany).

### Measurement of Airway Hyper-responsiveness

Airway hyper-responsiveness was measured by whole body plethysmography (Data Sciences International, New Brighton, MN, USA) (19). Mice received nebulized PBS, followed by increasing concentrations of methacholine (3.125, 6.25, 12.5, 25, and 50 mg/ml) to induce bronchoconstriction.

### Statistical Analyses

Data are presented as means alone or means ± SD. Two group comparisons were performed using Mann–Whitney *U*-tests. One-way analysis of variance with Tukey's multiple comparison tests was used to compare multiple groups. Statistical analyses were performed using Prism6 (Graph-Pad Software), and differences were considered statistically significant when *p* < 0.05 (indicated in figures as ^*^p < 0.05 and ^**^p < 0.01).

## Results

### RNA Virus Infections Induce dsDNA Release in the Lung

**Figure 1:**
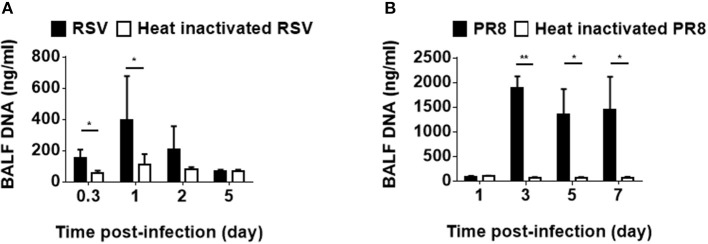
Host-derived dsDNA following infection with RNA viruses. Mice were intratracheally inoculated with RSV (A2) **(A)** or influenza virus (H1N1, A/PR8/34) **(B)**. Double-stranded DNA in the BALF was measured at various hours and days after infection. Three to nine mice were used per group. Data are presented as means ± SD. *p < 0.05, **p < 0.01.

Viral infections impact the host immune system not only by releasing viral pathogen-associated molecular patterns but also by leading host cells to release DAMPs, both of which have been shown to be involved in the induction and exacerbation of asthma (20). To examine viral infection induce host DNA as a part of DAMPs in infected lung tissue, mice were intratracheally inoculated with RSV, followed by BALF collection. Although RS virus is a RNA virus, dsDNA was detected in the BALF (Figure 1A). In addition, influenza virus infection also induced dsDNA in the BALF (Figure 1B). These results suggest that host dsDNA as DAMPs can be induced by RNA virus airway infections and might be associated with infection-induced lung inflammation and cell death, as well as the severity of these conditions.

### cGAMP Enhances Type-2 Immune Responses to Co-administered Antigen and the Allergic Inflammation

**Figure 2:**
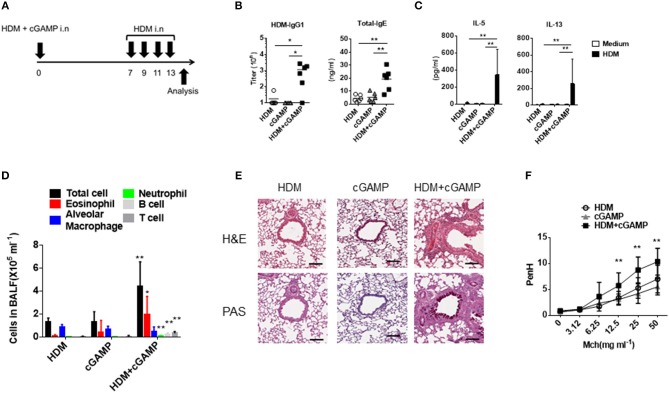
Type 2 immune responses and lung inflammation following cGAMP-induced allergic inflammation. **(A)** Experimental procedure for creating our cGAMP-induced allergic inflammation model. **(B)** Serum HDM-specific IgG1 and total IgE responses in mice sensitized with HDM, cGAMP, or HDM + cGAMP. **(C)** IL-5 and IL-13 production from lymph node cells in response to HDM. **(D)** The number of various immune cell populations found in the BALF after HDM challenge. *p < 0.05, **p < 0.01 are shown as comparison to both HDM and cGAMP **(E)** H&E and PAS staining of the lung sections. **(F)** Airway hyperactivity response as measured by enhanced pause (PenH) to methacholine (Mch). Six mice were used per group, and data represent one of two independent experiments with similar results. Data are presented as means ± SD. *p < 0.05, **p < 0.01.

Because cGAS binds dsDNA and generates the second messenger cGAMP (9), we utilized cGAMP as an alternative stimulus for DNA. To assess the impact of cGAMP in allergic inflammation in mice, we intranasally sensitized mice with HDM alone, cGAMP alone, or a combination of HDM and cGAMP (HDM + cGAMP), and 7 days later, we challenged the mice with HDM four times every other day (Figure 2A). On day 14, the mice sensitized with HDM + cGAMP had significantly higher serum levels of HDM-specific IgG1 and total IgE (Figure 2B), and HDM-specific Th2 cytokines in the lymph nodes (Figure 2C), than those sensitized with HDM alone or with cGAMP alone. When we examined the numbers of total cells, eosinophils, alveolar macrophages, neutrophil, B cells, and T cells in BALF, significantly higher numbers were observed in the mice sensitized with HDM + cGAMP than in the control mice (Figure 2D). The number of eosinophils recruited into the lung was dependent on the dose of cGAMP, suggesting that these responses were directly associated with the dose of cGAMP during the sensitization phase (Supplementary Figure 1A). Notably, there are two types of cGAMP, based on their structure. Mammalian cGAS produces 2′-3′ cGAMP, while bacterial cGAS produces 3′-3′ cGAMP. However, we obtained similar results using both types of cGAMPs (Supplementary Figure 2).

Histological analyses revealed severe inflammatory cell infiltration and goblet cell hyperplasia in the lungs of mice sensitized with HDM + cGAMP (Figure 2E). In addition, mice sensitized with HDM and cGAMP significantly increased the airway hyper-responsiveness to methacholine *in vivo* compared to the mice sensitized with HDM alone or treated with cGAMP alone (Figure 2F). Of note, we chose a relatively low dose (1 μg) of HDM for the sensitization because a higher dose of HDM induced inflammatory responses in the absence of cGAMP, while a singular administration of 1 μg of HDM failed to induce inflammatory responses in the absence of cGAMP (Supplementary Figure 1B). The allergic inflammation in the lung induced by repeated intranasal administration of high dose of HDM is known to be dependent on TLR4 (21), but our model likely differs from the conventional HDM-induced allergy model, as the allergic inflammation in our model was independent of TLR4 (Supplementary Figure 3). These results suggest that cGAMP can be one of the aggravating factors for HDM-induced allergic responses in the airway, especially under certain conditions in which DNA and cGAMP are physiologically relevant.

### IL-33 Is Required for cGAMP-Induced Allergic Inflammation in the Lung

**Figure 3:**
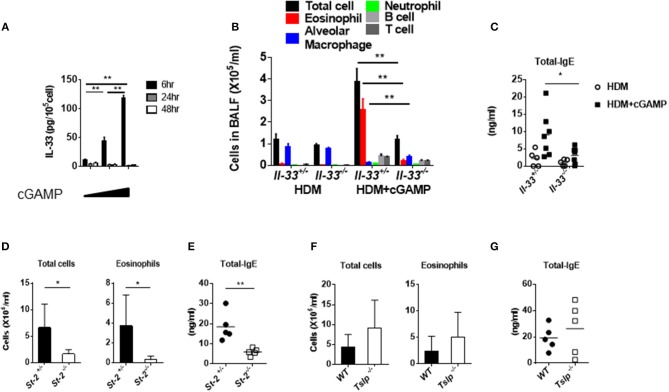
Assessment of IL-33 and acquired immunity during cGAMP-induced allergic inflammation in mouse lungs. **(A)** Intracellular IL-33 levels in cGAMP-treated (0, 1, 10 μg/ml) lung fibroblast cells. **(B–G)** Serum IgE levels and the numbers of cells in the BALF of wildtype or heterozygous mice compared with *Il-33*^−/−^ **(B,C)**, *St-2*^−/−^ **(D,E)**, or *Tslp*^−/−^ **(F,G)** mice that had been treated with HDM + cGAMP. Five to seven mice were used per group, and the data represent one of two independent experiments with similar results. Data are presented as means ± SD. *p < 0.05, **p < 0.01.

IL-33, IL-25, and TSLP are known to be pro-allergic cytokines, and they are released from various cells, including epithelial cells (22). Notably, IL-33 and ST2/IL1RL1 (IL-33 receptor) genes are considered to be asthma disease susceptibility genes and have been identified in genome-wide association studies (23). During lung inflammation, IL-33 is mainly released from pulmonary epithelial cells to elicit the recruitment and activation of immune cells that induce Th2 cytokines. These responses seem to be associated with the onset and disease pathologies of allergy (24). When we stimulated mouse lung fibroblast cells *in vitro* with cGAMP, the cell-associated IL-33, but not the IL-33 in the supernatant (data not shown), was highly upregulated within 6 h (Figure 3A). Given that cGAMP induces IL-33, we next investigated the role of IL-33 in cGAMP-adjuvanted, HDM-induced allergic inflammation using *Il-33*^−/−^ mice. Antibody responses and eosinophil recruitment into the lung were significantly decreased in *Il-33*^−/−^ mice compared with those in *Il-33*^+/−^ mice (Figures 3B,C). To further confirm the importance of IL-33, we carried out similar experiments using *St2*^−/−^ mice, which lack the IL-33 receptor, and found that, similar to *Il-33*^−/−^ mice, the antibody responses and eosinophil infiltration were significantly lower in *St2*^−/−^ mice than those in *St2*^+/−^ mice (Figures 3D,E). Although TSLP is known to be an important molecule in allergic inflammation, it is not involved in the antibody responses and inflammatory cell recruitment into the lung (Figures 3F,G). These results suggest that IL-33 and its receptor, ST2, are required for cGAMP-adjuvanted, HDM-induced allergic inflammation in mouse lungs.

### Acquired Immunity Is Required for cGAMP-Induced Allergic Inflammation in the Lung

**Figure 4:**
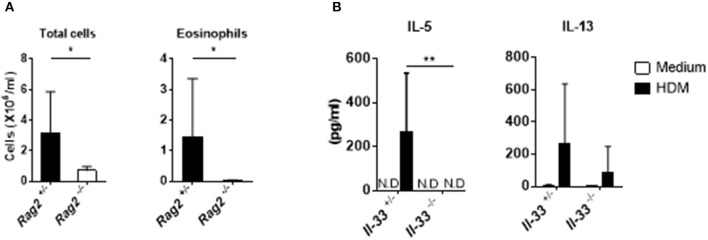
Acquired immunity during cGAMP-induced allergic inflammation in mouse lungs. **(A)** Numbers of total cells and eosinophils in BALF from *Rag2*^−/−^ mice. **(B)** IL-5 and IL-13 production in lymph node cells from *Il-33*^−/−^ mice. N.D. indicates that the level was below the limit of detection. Five to six mice were used per group, and the data represent one of two independent experiments with similar results. Data are presented as means ± SD. *p < 0.05, **p < 0.01.

IL-33 is a potent activator of group 2 innate lymphoid cells (ILC2) in the lung (25). Allergic inflammation induced by intranasal IL-33 administration is dependent on the activation of ILC2s but not on that of T or B cells, and IL-33 induced by papain stimulates strong airway eosinophilia in *Rag2*^−/−^ mice (26, 27). However, in our experimental system, the numbers of total cells and eosinophils in the BALF were significantly reduced in *Rag2*^−/−^ mice compared with those in *Rag2*^+/−^ mice (Figure 4A). This suggests that, unlike allergic inflammation induced by papain exposure, cGAMP-adjuvanted, HDM-induced allergic inflammation is mediated by adaptive T and/or B cells. To further confirm the role of IL-33 in acquired immunity in this allergic inflammation model, we next examined the HDM-specific cytokine production from the draining lymph nodes. As expected, cells from *Il-33*^−/−^ mice displayed lower levels of Th2 cytokines in response to HDM than cells from *Il-33*^+/−^ mice (Figure 4B), suggesting that acquired immune cells are necessary for cGAMP-adjuvanted, HDM-induced allergic inflammation in the lung.

### A TBK1 Inhibitor Improves cGAMP-Induced Allergic Inflammation in the Lung

**Figure 5:**
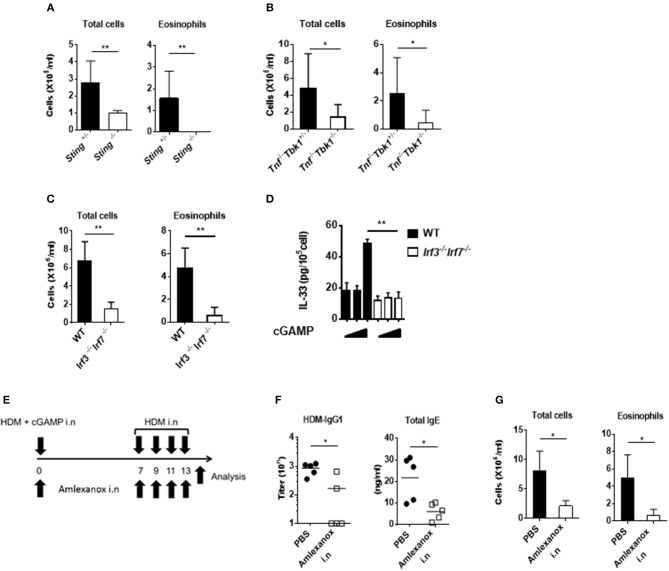
Effect of a TBK1 inhibitor on cGAMP-induced allergic inflammation in mouse lungs. **(A–C)** The numbers of total cells and eosinophils in BALF from *Sting*^−/−^ **(A)**, *Tnf*^−/−^*Tbk1*^−/−^ **(B)**, and *Irf3*^−/−^*Irf7*^−/−^ **(C)** mice. **(D)** Intracellular IL-33 levels at 6 h after stimulation in cGAMP-treated (0, 1, 10 μg/ml) lung fibroblast cells from wildtype and *Irf3*^−/−^*Irf7*^−/−^ mice. **(E)** Experimental procedure: mice were intranasally administered amlexanox (75 μg/dose, 25 mg/ml) or PBS applied together with HDM+ cGAMP at the exact time **(F,G)** Antibody responses **(F)** and inflammatory cells in BALF **(G)** of mice treated as shown in **(E)**. Five to six mice were used per group, and the data represent one of two independent experiments with similar results. Data are presented as means ± SD. *p < 0.05, **p < 0.01.

Because cGAMP is a ligand for STING and induces type-I interferon through TBK1 and IRF3 activation, we examined the importance of this signaling pathway for cGAMP-adjuvanted, HDM-induced allergic responses using *Sting*^−/−^, *Tbk1*^−/−^, and *Irf3*^−/−^*Irf7*^−/−^ mice. As expected, we observed that the amount of eosinophil recruitment into the lungs was significantly lower in all of the tested gene-deficient mice than that in the control mice (Figures 5A–C). Moreover, the intracellular IL-33 level in lung fibroblast cells stimulated by cGAMP was not induced in cells from *Irf3*^−/−^*Irf7*^−/−^ mice (Figure 5D).

Corticosteroids are key drugs for clinical asthma treatment (28), so we tested if corticosteroids were effective in our cGAMP-adjuvanted, HDM-induced allergic inflammation model. Dexamethasone (DEX) treatment during the elicitation phase decreased the number of eosinophils in the BALF compared with PBS treatment. Furthermore, histological analyses showed reduced goblet cell hyperplasia in the lung after DEX treatment compared with that after PBS treatment (Supplementary Figures 4A–C). Molecular-targeted therapy, such as anti-cytokine antibodies and anti-IgE antibodies, have recently been proposed for the treatment of allergic diseases (29, 30). Our results suggest that the STING-TBK1-IRF3 axis might be an important therapeutic target for cGAMP-induced allergic inflammation. Amlexanox is a small molecule and an active pharmaceutical ingredient of a drug used to treat asthma and allergic rhinitis in Japan. This drug functions as a TBK1 inhibitor (31). Therefore, we tested amlexanox as a molecular-targeted therapy drug in our model. Intranasal amlexanox administration during both the sensitization and elicitation phases significantly decreased inflammatory cell recruitment into the lung as well as the serum total IgE and HDM-specific IgG1 responses (Figures 5E–G). Similar results were observed in mice treated with amlexanox by daily gavage (Supplementary Figure 5). These results suggest that a TBK1 inhibitor can improve cGAMP-adjuvanted, HDM-induced asthma.

## Discussion

We used cGAMP as an endogenous adjuvant for the sensitization of allergic responses. This molecule was recently identified as a second messenger for DNA sensing. We previously reported that cGAMP and other STING ligands function as type 2 adjuvants, characterized by the induction of serum IgE levels, suggesting that cGAMP may be one of the endogenous factors (adjuvants) for the promotion of type 2 immune responses and allergic asthma. STING-mediated-type-2 immune response by B cells to the immunized Ag is dependent on both IRF3 and IFNαβR (12), but the Th2 response to the immunized Ag was not dependent on IFNαβR, while it was dependent on IRF3/7, STING and MyD88 (13), suggesting that there is IFNαβR-independent, MyD88-dependent activation pathway toward STING-mediated Th2 responses and consequent the lung asthmatic inflammation. Now in this study, we found that cGAMP functions as an allergy-prone adjuvant inducing strong type-2 immune responses to co-inhaled allergen in the airway which was dependent on IL-33 and its receptor ST2, which utilize MyD88-dependent signaling pathway.

A large number of previous studies have used mouse models to demonstrate causative factors that are involved in allergic asthma, and the model mice in many of these cases were prepared by intraperitoneal immunization with allergens in combination with alum. However, although this type of model mimics some of the pathological features seen in humans, such as eosinophil infiltration and serum IgE elevation, it does not reflect the physiological setting of allergic asthma because sensitization by intraperitoneal injection is an artificial route of immunization. In general, sensitizations to allergens occur in the airway or through the skin (32). Although, some studies have demonstrated pathogenesis in murine mouse models asthma model mice induced by the direct sensitization of allergens to the airway, frequent and large amounts of allergen administration to the airway are required for the preparation of this type of allergic asthma model (33). Furthermore, several studies concerning the direct sensitization of HDM have indicated that various factors, such as IL-21 from CD4^+^ T cells (34), IL-33 from inflammatory monocytes (35), TLR4-dependent inflammation, and IRF3 signals (21, 36), participate in mouse models of allergic asthma. In most cases, 10–100 μg of HDM were used for the induction of allergic inflammation; this differs from our model in which the mice received 1 μg of HDM. HDM also has proteolytic activity and induces a disruption of epithelial cell barrier function (37), indicating that high doses of HDM may cause host cell damage and/or death, which release DAMPs, including DNA. Indeed, we observed that sensitization with 5 μg of HDM induced allergic inflammation even in the absence of any adjuvant (cGAMP). Although, this low dose (1 μg) of HDM had no effect on the eosinophil activation and IgE responses, we found that cGAMP promoted allergic responses when administered with a low dose of HDM, suggesting that cGAMP might be an endogenous exacerbation factor for allergic asthma.

Interestingly, recent reports have shown that factors from dying cells, specifically DAMPs such as uric acid and ATP, function as adjuvants and exacerbate allergic inflammation (38, 39). These results suggest that endogenous factors with adjuvant activity might be involved in the sensitization of allergic diseases. In this study, we focused on an endogenous adjuvant for the induction of allergic asthma.

One recent report indicated that, in addition to being activated by infection with DNA viruses, the cGAS-cGAMP-STING axis is also activated by infection with RNA viruses (40). In fact, our results indicated that RNA virus infection leads to the release significant amount of DNA in the lung (Figure 1). This result suggests that the adjuvant activity of cGAMP might be involved in allergic asthma induced by an RNA virus, such as respiratory syncytial virus although we could not detect cGAMP in RSV infected lung homogenates (data not shown).

IL-33, a well-known DAMP (alarmin), is one of the key molecules for the pathogenesis of allergic asthma. It activates ILC2 cells to induce IL-5 and IL-13, and, in turn, these cells promote the recruitment of eosinophils and the induction of airway hypersensitivity (41, 42). Some allergens bearing protease activity, such as papain, mainly activate ILC2 cells through their enzymatic activity and induce eosinophilic inflammation. This type of inflammation is known to be stimulated by innate responses and is not mediated by acquired immunity (26, 27). In contrast, our model was completely dependent on acquired immune responses, as demonstrated by our finding that HDM + cGAMP-induced eosinophilic inflammation was abrogated in RAG2-deficient mice. We cannot rule out the role of ILC2 cells in our cGAMP-induced asthma model because ILC2 cells can participate in acquired immune responses in addition to their role in innate immune responses (43). Future experiments should investigate whether or not ILC2 cells are involved in the cGAMP-induced allergic asthma model.

Several drugs are used clinically in an effort to alleviate allergic inflammation, such as corticosteroids and leukotriene antagonists (44). These drugs are able to improve asthmatic symptoms in early childhood but do not alter asthma development (45). Amlexanox is a molecular-targeted drug with mechanism(s) of action is clearly different from those of corticosteroids and leukotriene antagonists. Specifically, amlexanox acts as a TBK1 inhibitor (31), and that TBK1 is a crucial factor for the induction of IL-33 (46). We also found that IRF3/7, which are signal transducers downstream of TBK1, are required for IL-33 release from lung fibroblasts in response to cGAMP. Together, these findings suggest that TBK1 inhibitors could be a promising class of anti-asthma drugs.

In conclusion, cGAMP functions as a type 2 adjuvant in the lung and contributes to exacerbation of asthma. STING/TBK1/IRF3 axis and IL-33 signaling, required for cGAMP-induced allergic inflammation, may be novel therapeutic targets for allergic asthma.

## Data Availability

All datasets generated for this study are included in the manuscript/Supplementary Files.

## Ethics Statement

The animal study was reviewed and approved by National Institute of Biomedical Innovation, Health and Nutrition (NIBIOHN).

## Author Contributions

KO, BT, TK, SK, MM, KK, and EK performed most experiments. CC, EK, SI, and KI supervised the study. KO, EK, and KI wrote the manuscript. All authors approved the final version.

### Conflict of Interest Statement

The authors declare that the research was conducted in the absence of any commercial or financial relationships that could be construed as a potential conflict of interest.

## Abbreviations

BALF: Broncho-alveolar lavage fluid
cGAMP: Cyclic GMP-AMP
DAMPs: Damage-associated molecular patterns
HDM: House dust mite antigen
ILC2: Group 2 innate lymphoid cell
IRF: Interferon regulatory factor
STING: Stimulator of IFN genes
TBK-1: TANK-binding kinase 1
TSLP: Thymic stromal lymphopoietin
DEX: Dexamethasone.
