# Supplementary material for: Cyclic GMP-AMP Triggers Asthma in an IL-33-Dependent Manner That Is Blocked by Amlexanox, a TBK1 Inhibitor
Source: Front Immunol. 2019 Sep 26;10:2212. doi: 10.3389/fimmu.2019.02212 (PMC6775192; doi:10.3389/fimmu.2019.02212)
Supplement: Supplementary file 1 [file Data_Sheet_1.pdf]

Supplementary figure 1

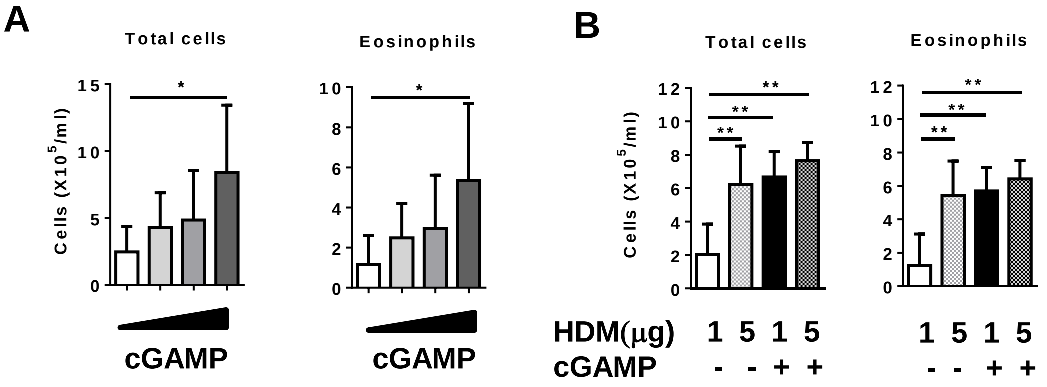

Supplementary Figure 1. Dose response of cGAMP and HDM on inflammatory cell recruitment into the lung.
(A) Numbers of total cells and eosinophils in the BALF of mice sensitized with different amounts of cGAMP (0, 0.1, 0.3, or 1.0 μg/ml) and HDM. (B) Mice were sensitized intranasally with HDM in the presence or absence of cGAMP, and then mice were challenged intranasally with HDM four times. The numbers of total cells and eosinophils in the BALF in these mice are shown. Four or six mice were used per group, and the data represents one of two independent experiments with similar results. Data are presented as means ± SD. *$p < 0.05$, **$p < 0.01$.

Supplementary figure 2

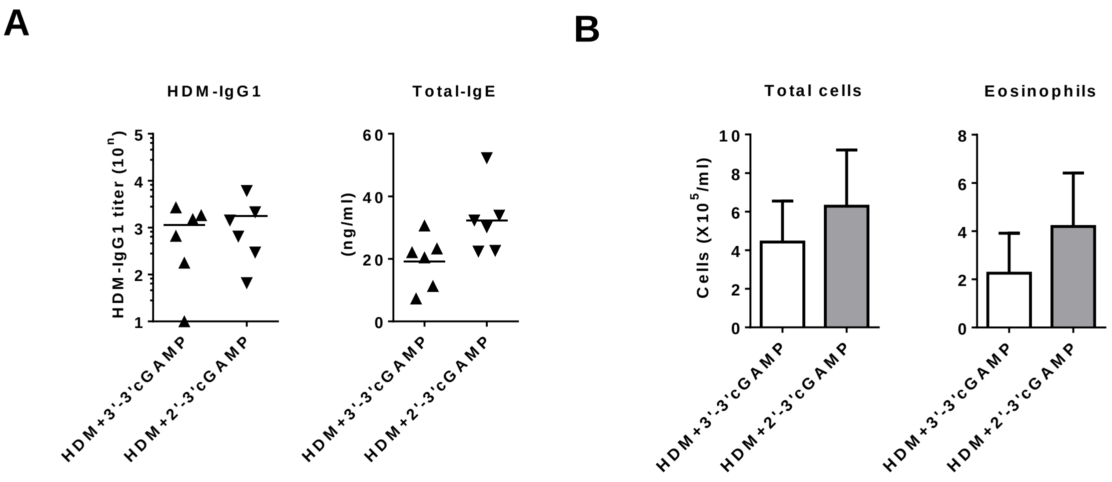

Supplementary Figure 2. Effects of 2′-3′ cGAMP and 3′-3′ cGAMP on allergic responses.

Mice were treated with 2′-3′ cGAMP or 3′-3′ cGAMP. Serum antibody responses (A) and the numbers of total cells and eosinophils in BALF (B) from these mice are shown. Six mice were used per group, and the data represents one of two independent experiments with similar results. Data are presented as means ± SD. *p < 0.05, **p < 0.01.

Supplementary figure 3

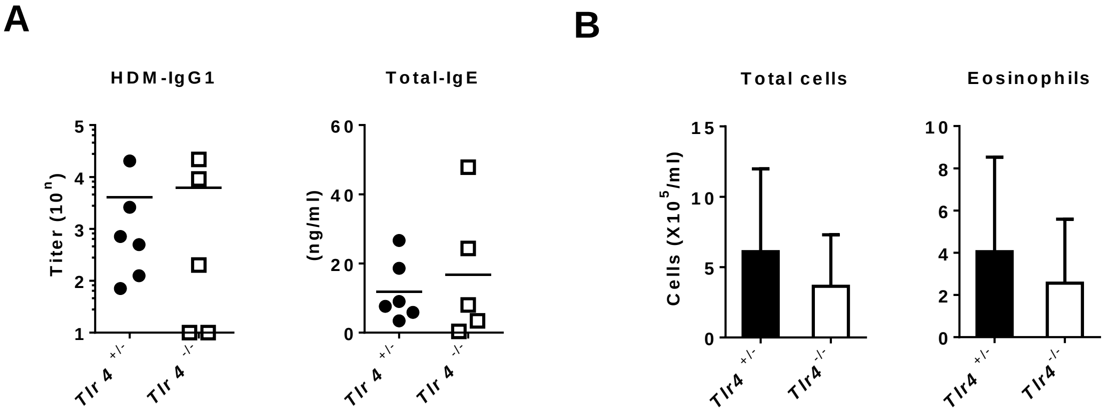

Supplementary Figure 3. Assessment of TLR4 involvement in cGAMP-induced allergic inflammation.

Tlr4+/− and Tlr4−/− mice were intranasally immunized with HDM + cGAMP and then challenged with HDM four times (at days 7, 9, 11, and 13). Serum antibody responses (A) and the numbers of total cells and eosinophils in BALF (B) are shown. Five or six mice were used per group, and the data represents one of two independent experiments with similar results. Data are presented as means ± SD. *p < 0.05, **p < 0.01.

Supplementary figure 4

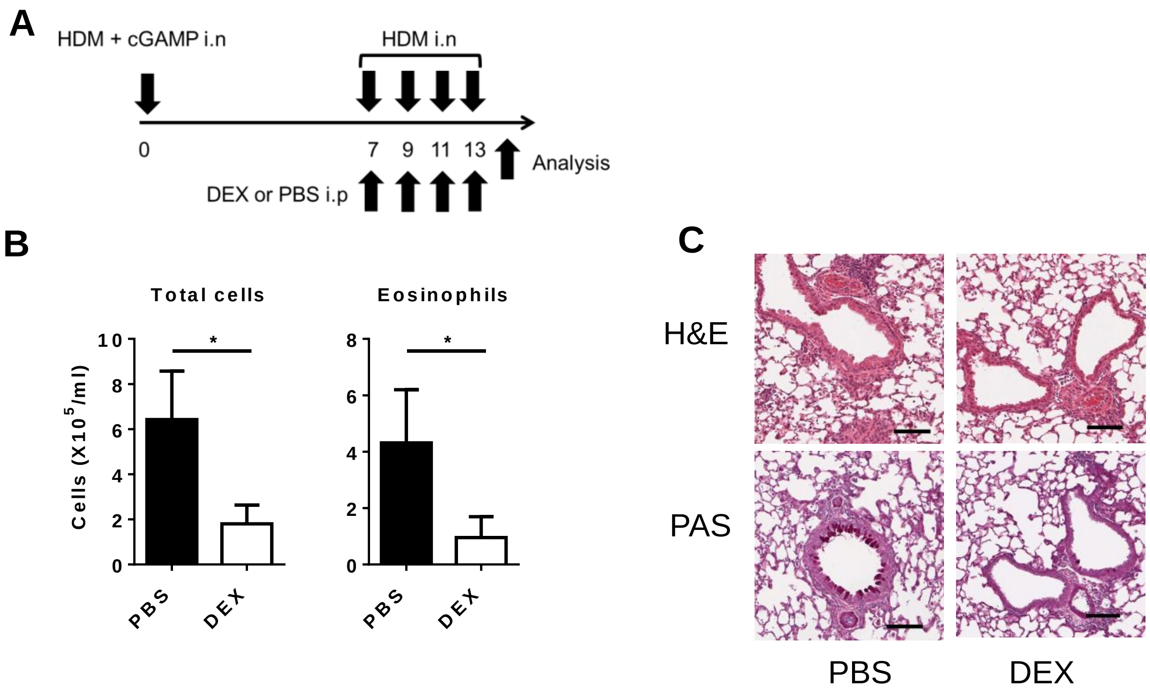

Supplementary Figure 4. Effect of dexamethasone (DEX) on cGAMP-induced allergic inflammation in mouse lungs.

(A) Experimental procedure of DEX treatment in our cGAMP-induced allergic inflammation model. DEX (5 mg/kg) or PBS was intraperitoneally (i.p.) injected 1 h before intranasal (i.n.) HDM challenges. (B) Numbers of total cells and eosinophils in BALF after DEX or PBS treatment. (C) H&E and PAS staining of the lung sections from mice after DEX or PBS treatment. Four or five mice were used per group, and the data represents one of two independent experiments with similar results. Scale bar, 100μm. Data are presented as means ± SD. *$p < 0.05$, **$p < 0.01$.

Supplementary figure 5

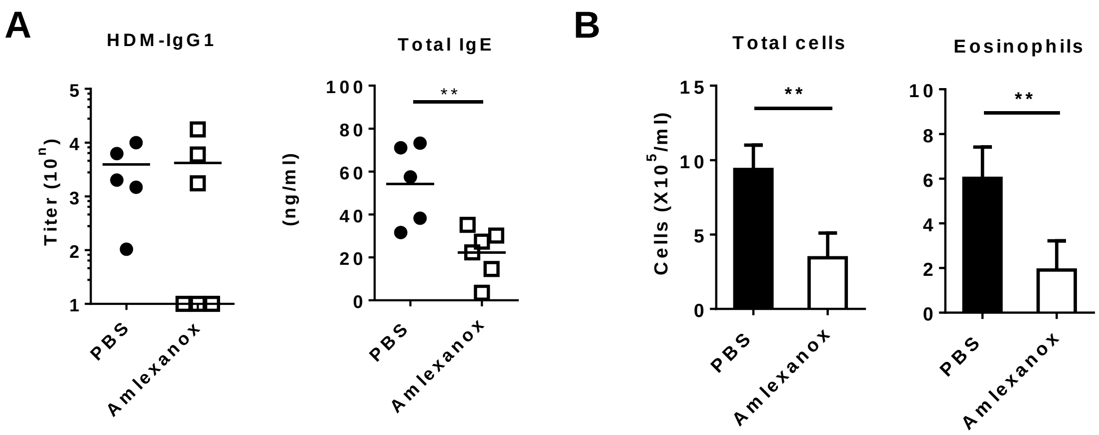

Supplementary Figure 5. Effect of oral administration of a TBK1 inhibitor on cGAMP-induced allergic inflammation in the lung.

Wildtype mice that had been sensitized with HDM + cGAMP were treated with amlexanox (100 mg/kg) or PBS by daily gavage until day 14. The serum antibody response (A) and the numbers of total cells and eosinophils in the BALF (B) of these mice are shown. Five or six mice were used per group, and the data represents one of two independent experiments with similar results. Data are presented as means ± SD. *$p < 0.05$, **$p < 0.01$.
